# Impaired Self-Other Distinction and Subcortical Gray-Matter Alterations Characterize Socio-Cognitive Disturbances in Multiple Sclerosis

**DOI:** 10.3389/fneur.2019.00525

**Published:** 2019-05-21

**Authors:** Kristína Czekóová, Daniel Joel Shaw, Kristína Saxunová, Michal Dufek, Radek Mareček, Jiří Vaníček, Milan Brázdil

**Affiliations:** ^1^Behavioral and Social Neuroscience, Central European Institute of Technology (CEITEC), Masaryk University, Brno, Czechia; ^2^Institute of Psychology, Czech Academy of Sciences, Brno, Czechia; ^3^Department of Psychology, School of Life and Health Sciences, Aston University, Birmingham, United Kingdom; ^4^First Department of Neurology, Faculty of Medicine, Masaryk University and St. Anne's University Hospital, Brno, Czechia; ^5^Multimodal and Functional Neuroimaging, Central European Institute of Technology (CEITEC), Masaryk University, Brno, Czechia; ^6^Department of Imaging Methods, Masaryk University and St. Anne's University Hospital, Brno, Czechia

**Keywords:** multiple sclerosis, social cognition, self-other distinction, automatic imitation, visual perspective taking, voxel-based morphometry, gray-matter volume

## Abstract

**Introduction:** Recent studies of patients with multiple sclerosis (MS) have revealed disturbances in distinct components of social cognition, such as impaired mentalizing and empathy. The present study investigated this socio-cognitive profile in MS patients in more detail, by examining their performance on tasks measuring more fundamental components of social cognition and any associated disruptions to gray-matter volume (GMV).

**Methods:** We compared 43 patients with relapse-remitting MS with 43 age- and sex-matched healthy controls (HCs) on clinical characteristics (depression, fatigue), cognitive processing speed, and three aspects of low-level social cognition; specifically, imitative tendencies, visual perspective taking, and emotion recognition. Using voxel-based morphometry, we then explored relationships between GMV and these clinical and behavioral measures.

**Results:** Patients exhibited significantly slower processing speed, poorer perspective taking, and less imitation compared with HCs. These impairments were related to reduced GMV throughout the putamen, thalami, and anterior insula, predominantly in the left hemisphere. Surprisingly, differences between the groups in emotion recognition were not significant.

**Conclusion:** Less imitation and poorer perspective taking indicate a cognitive self-bias when faced with conflicting self- and other-representations. This suggests that impaired self-other distinction, and an associated subcortical pattern of GM atrophy, might underlie the socio-cognitive disturbances observed in MS.

## Introduction

Multiple sclerosis (MS) is a chronic demyelinating disease of the central nervous system. As part of a complex neurological symptomatology, MS patients present frequently with disturbances in various aspects of cognitive functioning; slower processing speed, impaired episodic memory and executive function are among the most affected (1, 2). Another domain in which dysfunction manifests is social cognition (3)—that is, the broad repertoire of cognitive and affective skills that allow us to infer others' mental and emotional states in order to interact with them effectively (4, 5). Patients' quality of life is reduced considerably by disruptions to their social environment, and so the development of effective interventions requires a better understanding of the socio-cognitive deficits that impact negatively on their interpersonal relationships. Since the range of abilities comprising social cognition span various levels of complexity, however, a precise characterization of these impairments is difficult with standard neuropsychological tests (6).

Unlike the long history of research into general cognitive impairments in MS, only recently have studies begun to unveil the nuanced nature of disturbances in social cognitive abilities exhibited by these patients. The findings of these studies often converge to reveal difficulties in lower-level capacities, particularly in the recognition of negatively valenced facial emotions [e.g., sadness, anger, fear; (7)]. In contrast, investigations of other higher-level components of social cognition provide less consistent insights; while some report that patients are impaired in their ability to attribute mental (“mentalizing”) *and* affective states to others [“empathy”; (8, 9)], other studies have observed disturbances only in cognitive mentalizing (10, 11). Moreover, it remains to be seen whether impairments in these high-level facets of social cognition result from disruptions to more fundamental components.

Although the precise structure of social cognition is yet to be defined, current research suggests a hierarchical organization in which lower-level mechanisms contribute to or provide a necessary prerequisite for higher-level processes (4). In this model, mentalizing is believed to build upon a more fundamental ability to take another's perspective. Consistent with this notion, both perspective taking and mentalizing engage the superior temporal/temporo-parietal cortices [e.g., (12)]. Similarly, emotion recognition is considered a necessary prerequisite of empathy, and both processes are associated frequently with brain responses within the insulae and anterior cingulate cortex (13–15). More recently, we have shown that empathic expression is mediated by imitative tendencies, suggesting that a process of emotional simulation might be necessary for empathy (16).

Furthermore, multiple components of social cognition are thought to recruit a common mechanism of *self-other distinction* (SOD), which enables us to treat independently and distinguish flexibly between cognitive self and other representations (15, 17). Without efficient SOD, we might egocentrically misattribute our own cognitive and affective states onto others. This would be evident particularly when our own self states are incongruent with those of others (18), resulting in poor emotion recognition and empathic awareness, and inappropriate responding in social situations. Since the viewpoints of other individuals often conflict with our own, this mechanism is also necessary when inferring our interaction partners' perspective—adopting another's perspective requires us to detach ourselves from our own representations (19). On the other hand, dysfunction to this low-level cognitive mechanism might result in uncontrolled self-other merging. Humans exhibit an involuntary tendency to mimic one another during social interaction (20), which appears to reflect a common neural coding of self- and other-action [e.g., (21)]. Control of imitative tendencies therefore requires SOD to differentiate between our own and others' actions (17, 22); without this mechanism, we might exhibit hyperimitation [see (17)]. Consistent with the notion of SOD providing a mechanism common to both perspective taking and mimicry, past research has demonstrated that the expression of involuntary imitation is related inversely to perspective-taking performance (23, 24). Moreover, SOD is associated frequently with brain activity in the temporo-parietal cortices (15). Disturbances to SOD might therefore underlie the higher-level socio-cognitive deficits observed in MS.

Traditionally, MS has been characterized in terms of white matter (WM) pathology, but recent research indicates that gray matter (GM) abnormalities can predict dysfunctional social cognition in this patient population (25). GM atrophy within deep nuclei and the limbic system is present in the very early stages of MS (26), and progresses rapidly in all MS phenotypes (27). This is observed in the thalamus, putamen, caudate nucleus, globus pallidus, and amygdala (26, 28). While other brain regions are associated more frequently with social cognition, these subcortical structures do appear to play an important role in socio-cognitive functioning; the limbic and paralimbic system (including amygdala, striatum, temporal pole, and anterior cingulate) have been implicated in representation of self and other mental states, for instance, and the dorsal striatum has been associated with cognitive mentalizing (29). Correspondingly, disturbances in social cognition also comprise the symptomatology of Parkinson's and Huntington's disease—disorders characterized partly by disruptions to cortico-basal ganglia-thalamo-cortical circuits [e.g., (30)]. Focal GM atrophy among these structures might therefore contribute to the deficits in social cognition exhibited in MS. Unfortunately, however, the majority of research in MS has been performed exclusively at the behavioral or self-report level (8, 9, 11, 31), with relatively few studies combining this with neuroimaging data (25, 32, 33).

To achieve a better characterization of the disturbances in social cognition exhibited by MS patients, the present study utilized three experimental tasks designed to measure discrete, low-level socio-cognitive capacities; specifically, in line with the model described above (4) we measured emotion recognition, visual perspective taking, and imitative tendencies. To assess perspective taking, we measured patients' performance on a task that required them to infer other person's viewpoint when it is incongruent with their own. To examine emotion recognition we assessed their ability to infer the emotional state of another person from just their eyes. Finally, to quantify imitative tendencies we measured the degree to which they imitated the actions of another person automatically, even when this interfered with another task. Using GM volume (GMV) as a metric of brain structure, we then applied voxel-based morphometry to examine whether any of these behavioral indices of social cognition were related to patterns of neural atrophy. Given that MS is associated frequently with a pattern of GM atrophy throughout deep subcortical nuclei, and the apparent role of these nuclei in social cognition, we hypothesized that the degree of GM reduction throughout subcortical brain structures would be related positively to disruptions of SOD.

## Materials and Methods

### Sample

We recruited 43 patients with relapsing-remitting MS consecutively from the Department of Neurology at St. Anne's University Hospital, Czech Republic, and 43 healthy controls (HCs) matched on age, sex, and handedness (for details on demographics, see Table 1). Handedness was assessed with the revised version of the Edinburgh Handedness Inventory (34); a laterality quotient (LQ) was calculated as (right–left)/(right + left) × 100. Physical disability was assessed in MS patients with the Expanded Disability Status Scale [EDSS; (35)]. All patients had been diagnosed according to the revised McDonald criteria (36), and had no other neurological or psychiatric diagnoses. Patients reporting mild to moderate depressive symptoms were included in the study, but the extent of symptoms reported by the two groups were not statistically different (see below). The minimum duration in education was 12 years (i.e., completion of secondary education). Importantly, the availability of disease-modifying treatment for MS patients in the Czech Republic is currently limited to those meeting certain criteria. For this reason, only 36 of these asymptomatic patients (84%) were undergoing treatment: interferon beta 1a (*n* = 9), interferon beta 1b (*n* = 3), fingolimod (*n* = 6), glatiramer acetate (*n* = 6), dimethyl fumarate (*n* = 6), teriflunomide (*n* = 3), natalizumab (*n* = 2), and daklizumab (*n* = 1). The experiment was approved by the Institutional Review Board of St. Anne's University Hospital, and all individuals provided written informed consent prior to participating.

**Table 1:** Sample demographics and clinical characteristics.

	**Groups**		
	**MS**	**HC**	***P***
Age (mean [SD])	35.8 (8.0)	34.7 (11.0)	0.585
Males	12 (28 %)	18 (42 %)	0.175
EDSS (median [range])	2.5 (6)	–	–
DD (mean [SD])	7.5 (4.4)	–	–
LQ (median [range])	100 (120)	100 (200)	0.177
University degree	24 (56%)	43 (100%)	<0.001

### Procedure

Participants underwent behavioral assessment prior to brain scanning, which took place no longer than seven months afterwards (M = 5.2 months; SD = 1.6). Importantly, no relapses were presented during these examination periods. The test battery was performed in a single session lasting ~1 h, with each assessment administered in the order in which they are described below. Implicit task-performance measures were obtained before explicit and self-report assessments so that the latter could not influence the former.

### Cognitive Processing Speed

To screen for possible cognitive impairment, we employed a paper version of the Symbol Digit Modalities Test [SDMT; (37)]. This test has been established as a reliable and valid measure of cognitive processing speed in MS patients, and the best predictor of cognitive dysfunction in this population given the influence of processing speed on other cognitive functions (38, 39).

### Imitative Tendencies

**Figure 1:**
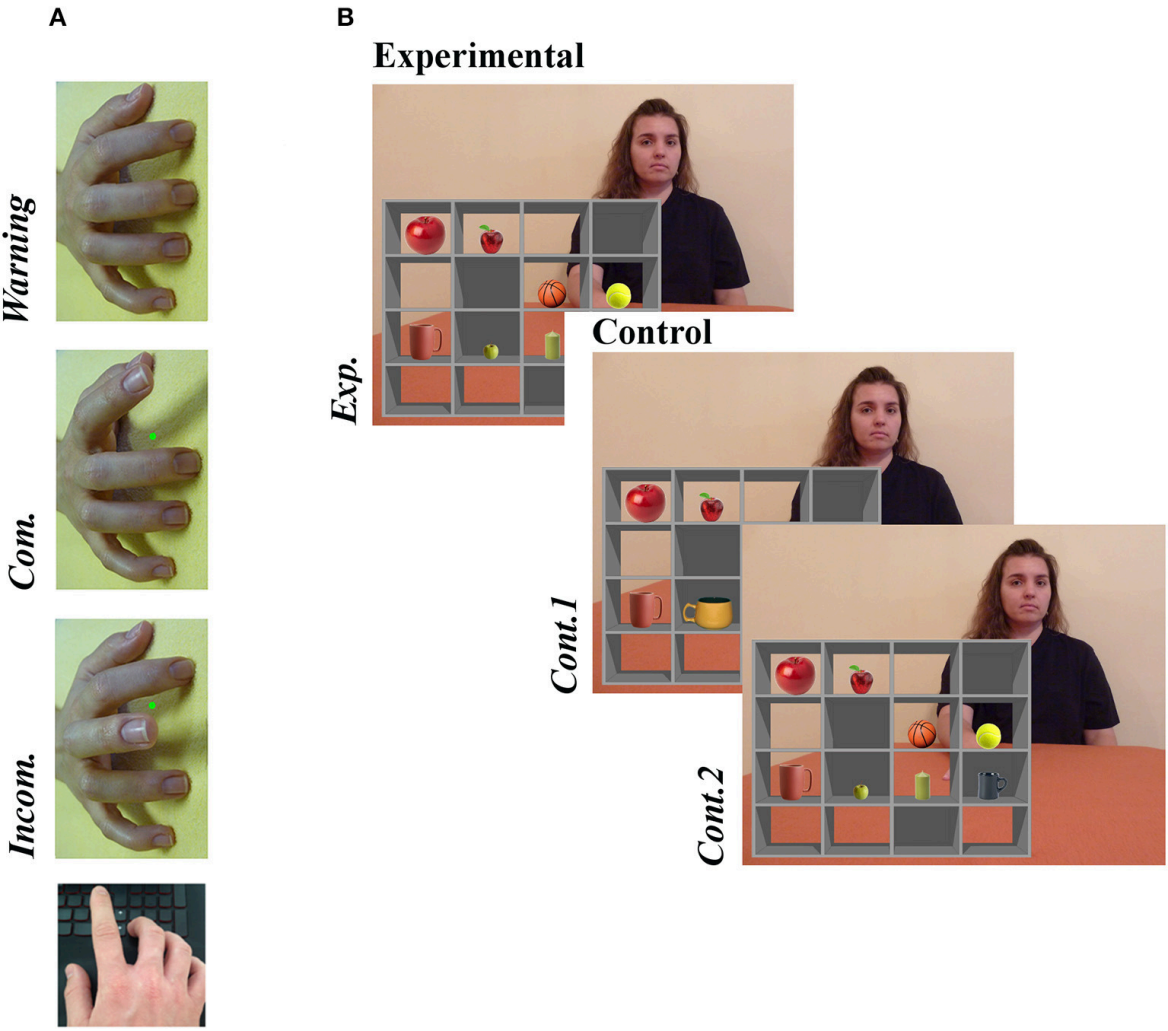
Examples of experimental stimuli for **(A)** the Stimulus-response compatibility procedure and **(B)** the Director Task. **(A)** A right stimulus hand was presented at a 90° counter-clockwise rotation. In this example, a green dot (imperative stimulus) signals that the participant should lift index finger. Whether the observed finger movement was the same or different to the one signaled by the imperative stimulus defined the condition (Compatible [*Com*.] or Incompatible [*Incom*.], respectively). **(B)** An audible instruction given by the Director asked the participant to move one of the items to a new box (“*Move the smallest apple one box down”*). On experimental trials (*Exp*.), the instruction referred to an object that created a discrepancy between the Director's and participants' perspectives, while no such discrepancy existed on control trials; no distractor object was present in *Cont. 1*, and in *Cont. 2* the instruction referred to a different object (“*Move the biggest apple one box down”*). Written informed consent was obtained from the individual for the publication of this image. For full instructions, see the Supplementary Information.

To measure imitative tendencies we employed a computerized stimulus–response compatibility procedure (40), whereby participants are required to execute finger-lifting actions in response to a colored dot (imperative stimulus) while observing task-irrelevant finger actions performed by a stimulus hand (Figure 1A). The degree to which participants are faster and more accurate at executing finger movements signaled by the imperative stimulus when they observe simultaneous matching (compatible) compared with opposing (incompatible) movements is referred to as automatic imitation, and is considered an experimental measure of spontaneous mimicry; higher scores represent greater imitative tendencies. Importantly, Genschow et al. (41) report high split-half reliability (0.86) for this compatibility effect.

All trials began with a warning stimulus, comprising a model's pronated right hand with all fingers resting on a flat surface but rotated 90° counter-clockwise from the participants' perspective. At this point, the participant depressed both the left and right directional arrows on a standard keyboard with the index and middle finger of their right hand, respectively. After 800, 1,600, or 2,400 ms, selected randomly, the warning stimulus was replaced with an end-point image of the same stimulus hand performing either an index or middle finger extension. In this end-point image, a red or green dot was presented between the index and middle finger. The color of this dot signaled whether the participant should move their own index or middle finger (e.g., green = index finger, red = middle finger; the color-finger pairing was counterbalanced across participants). In response to the imperative stimulus, the participant lifted the corresponding finger as quickly as possible, thereby releasing a key. The trial then ended with a blank screen lasting 1,000 ms. The task consisted of three trial types: compatible (the same finger action was signaled and observed), incompatible (opposite finger actions were signaled and observed), and baseline (movement was signaled but not observed). Overall, the paradigm comprised 70 trials−30 compatible, 30 incompatible, and 10 baseline trials—with accuracy and response time (RT) measured on each trial. It is important to emphasize that we used a right stimulus hand rotated 90° counter-clockwise. Since participants responded with their right hands, this stimulus isolated imitative- from any confounding spatial-compatibility effects to provide a pure measure of imitative tendencies (24).

### Perspective Taking

The Director task was used to assess individuals' ability to differentiate between their own visual perspective and that of another when the two viewpoints conflict [visual perspective taking; e.g., (42)]. Recently we observed that both RTs and accuracy demonstrated excellent split-half reliability in each condition [> 0.96; (16)].

As illustrated in Figure 1B, the stimulus consisted of a grid of shelves forming 16 boxes, with a different object placed in each of eight boxes. On each trial, the participant received a recorded verbal instruction from a female “Director” to move one of the objects to a different box. The Director sat behind the shelves and therefore could not see the contents of five boxes with opaque backs, which were visible only from the participant's (front) perspective. On experimental trials, the instruction referred to an object that created a discrepancy between the Director's and participants' perspectives (e.g., “*Move the smallest apple one box down,”* when the director could see only the medium-sized apple). To perform the instruction correctly on these experimental trials, the participant had to discount any “distractor” objects not visible to the Director (e.g., they were required to move the medium-sized apple rather than the smallest). In both control conditions, there was no conflicting object to discount: the distractor was replaced with a non-conflicting object in the first control condition (Cont.1), and in the second (Cont.2) the Director's instruction changed so as to render the distractor irrelevant (e.g., “*Move the biggest apple one box down*”). Each condition comprised 20 trials presented randomly. The audio recordings of instructions were equivalent across all trials [mean 3.26 (SD .22) sec]. Participants responded by clicking with the mouse on the box where the object should be moved. Errors involved selection of the wrong (e.g., distractor) object or wrong location, the latter including omission of left–right switching (moving the target object left one box when they were instructed to move it rightwards, or vice versa). Any potential difference in perspectives was emphasized on practice trials that included a front and rear view of the shelves (see Supplementary Material for instructions given to participants).

### Emotion Recognition

A paper version of the Reading the Mind in the Eyes Test [RMET; (43)] was employed to measure participants' ability to infer the emotional state of others. Although the task is employed frequently as a measure of affective mentalizing, it is considered by some scholars to measure only the first stage of this process—emotion recognition (44). This task contains 36 images depicting the eye region of actors' emotional facial expressions. Facial expressions represent complex emotional states with positive (e.g., playful, interested), negative (e.g., hostile, suspicious), and neutral valence (e.g., reflective, pensive). Each image is presented sequentially, and participants are required to select one of four labels that best match the expression without a time limit. A sum of correct responses is used as a measure of success.

### Depression

All participants completed the Beck Depression Inventory [BDI-II; (45)]. Using 21 items, this self-report instrument assesses cognitive, affective, physiological and motivational symptoms of depression experienced over the preceding 2 weeks. Scores of 20–28 indicate moderate depression. The BDI-II has been found to be a valid and reliable instrument for the evaluation of depressive symptoms in MS (46).

### Fatigue

Patients and controls completed the Modified Fatigue Impact Scale [MFIS; (47)], a self-report instrument that assesses the degree to which physical, cognitive, and psychosocial fatigue experienced over the preceding 4 weeks has affected every-day functions.

### MRI Acquisition

High-resolution T1-weighted anatomical images were acquired on a 1.5T Siemens Symphony scanner, using a standard 32-channel array head coil and an MPRAGE sequence: 176 sagittal slices (slice thickness = 1.17 mm); TR = 1,700 ms, TE = 3.93 ms, TI = 1,100 ms, flip angle = 15°; in plane matrix size 256 × 256, resampled to 512 × 512, FOV = 246 × 246 mm, in-plane resolution = 0.48 × 0.48 mm.

### Statistical Analyses

#### Behavioral Data

Differences between the groups were assessed using parametric or non-parametric *t*-tests, depending on the normality of variable distributions. Independent-samples *t*- and Mann-Whitney *U*-tests were employed to contrast MS patients with HCs. Since normality was violated for the majority of the variables, associations between them were examined by Spearman correlation coefficients, all of which were entered into a multivariate bootstrapping procedure (1,000 iterations) to obtain 95% confidence intervals (CIs). These intervals provide an estimate of population values for each coefficient, providing an alternative measure of significance; CIs including zero should be considered unreliable.

For calculating measures of imitative tendencies and perspective taking, we employed an approach that we have used previously to investigate relationships between these two components of social cognition (16, 24). The strength of imitative tendencies was expressed as the difference in response time (RT) between the incompatible relative to the compatible condition, and perspective-taking performance was expressed as the difference in RT and accuracy on the experimental relative to the control conditions. Importantly, there was no evidence of a speed-accuracy trade-off for perspective taking in this sample (*p* = 0.165), so relative measures for RT and accuracy scores were calculated separately. For both measures, responses on the control condition were regressed from those in the corresponding experimental condition(s), resulting in residualized scores that reflect the difference between the conditions: specifically, greater residuals reflect poorer performance (slower RTs and poorer accuracy in the experimental relative to the control conditions. It is important to emphasize that measures of both imitation and perspective taking are relative (RTs on incompatible *vs*. compatible trials, and experimental *vs*. control trials, respectively), and should therefore be uninfluenced by any differences in processing speed between MS patients and HCs. The statistical analyses were performed using SPSS 24 software.

#### Neuroimaging Data

To compare GMV between the brains of HCs and MS patients we analyzed MR images with the optimized VBM pipeline provided in FSL (48). This analysis pipeline produces results that converge closely with those from the Statistical Parametric Mapping platform (49).

First, the anatomical images were brain-extracted and segmented into GM, WM and cerebrospinal fluid using *FAST* (50), and the resulting GM partial-volume maps were affine-registered to the MNI-152 standard space template using *FLIRT* (51). The registered GM images from the entire sample (both HCs and MS patients) were then concatenated and averaged, and flipped along the x-axis. By re-averaging each mirror image to the MNI-152 template, a first-pass left-right symmetric, study-specific “affine” GM template was created. This step avoids introducing any bias during the registration process. Second, all native GM images were re-registered non-linearly to the affine template with *FNIRT* (52), concatenated, averaged, and flipped along the x-axis. Symmetric, study-specific “non-linear” GM templates were then created by averaging both mirror images, and native GM partial-volume maps were registered to their corresponding non-linear template. Importantly, this optimized protocol modulates each registered GM image to compensate for any contraction/enlargement due to the non-linear transformation; specifically, each voxel of each image was multiplied by the Jacobian of the warp field [see (53)]. Since this modulation does not include the affine part of the registration, however, no correction for total intracranial volume is needed (48). The modulated GM images were then smoothed with an isotropic Gaussian kernel with a sigma of 3 mm.

General Linear Modeling (GLM) was then applied to the resampled, smoothed and modulated GM images to assess localized differences between the HC and MS group. Since MS is characterized by localized WM lesions, appearing as hypointensities on T1 images that can result in an overestimation of GMV, we added to these group comparisons a covariate of no interest representing subject-specific values of mean WM calculated from the corresponding partial-volume map. Subsequently, by adding measures from patients' clinical assessment or behavior on each experimental task as covariate regressors in further GLM analyses, we examined whether localized GMV in the MS group was related to clinical characteristics or socio-cognitive performance. Using *randomize* (37), all resulting statistical maps were thresholded with permutation-based non-parametric inference; 5,000 permutations were performed with threshold-free cluster enhancement (54), and family-wise error (FWE)-corrected for multiple comparisons.

## Results

The values below present means (±SD).

### Clinical Assessment

**Figure 2:**
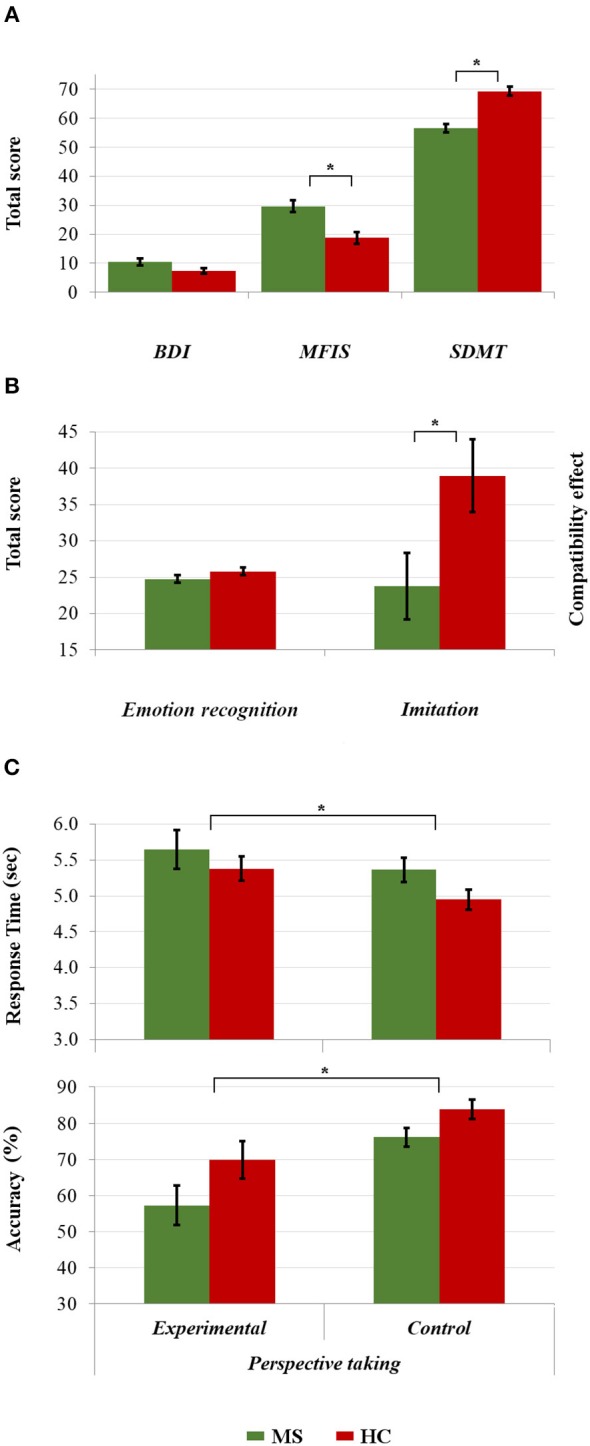
Comparisons between the HC and MS group in **(A)** clinical assessment and cognitive processing speed, **(B)** emotion recognition and imitative tendencies, and **(C)** response time and accuracy in visual perspective taking. *p < 0.05.

The MS patients performed worse than the HCs on the SDMT (56.42 [±9.26] vs. 69.21 [±10.00]; *t*_(84)_ = 6.154; *p* < 0.001, *d* = 1.33), and reported greater fatigue on the MFIS (29.65 [±12.99] vs. 18.72 [±13.59]; *U* = 518.00; *p* < 0.001; *r* = 0.38). Although the MS group also expressed more depression (10.53 [±8.20] vs. 7.35 [±6.06]), this difference was not statistically significant (*p* = 0.055; *r* = 0.21; see Figure 2A).

### Behavioral Performance

Five MS patients and three HCs were excluded from the analyses of perspective-taking performance because they achieved a score of zero in the experimental condition of the Director Task, suggesting a misunderstanding of task instructions. Both imitation and perspective taking differed between the groups: compared with HCs, patients showed significantly less imitation (23.78 [±30.22] vs. 38.97 [±32.89] ms; *t*_(84)_ = 2.23, *p* = 0.028; *d* = 0.48), and both longer response time (0.21 [±1.14] vs. −0.20 [±0.79]; *U* = 525.00, *p* = 0.019; *r* = 0.27) and poorer accuracy in perspective taking (−0.20 [±0.97] *vs. 0*.20 [±0.99]; *U* = 649.00, *p* = 0.017, *r* = 0.26). Surprisingly, however, emotion recognition was similar in the MS patients and HCs (24.74 [±3.44] vs. 25.81 [±3.57]); *U* = 731.50, *p* = 0.094; *r* = 0.18; See Figures 2B,C).

Correlations between the clinical assessments and socio-cognitive measures revealed significant relationships only between disease duration and accuracy in perspective taking (ρ_(36)_ = −0.36; *p* = 0.026; CI = [−0.62, −0.06]). No associations emerged with respect to other measures of social cognition (*p* ≥ 0.086), or self-reported fatigue and depression (*p* ≥ 0.257; see Table S1). As expected, there were no significant relationships between cognitive processing speed (SDMT scores) and any measure of socio-cognitive performance (*p* ≥ 0.217, see Table S2).

### Neuroanatomy

**Figure 3:**
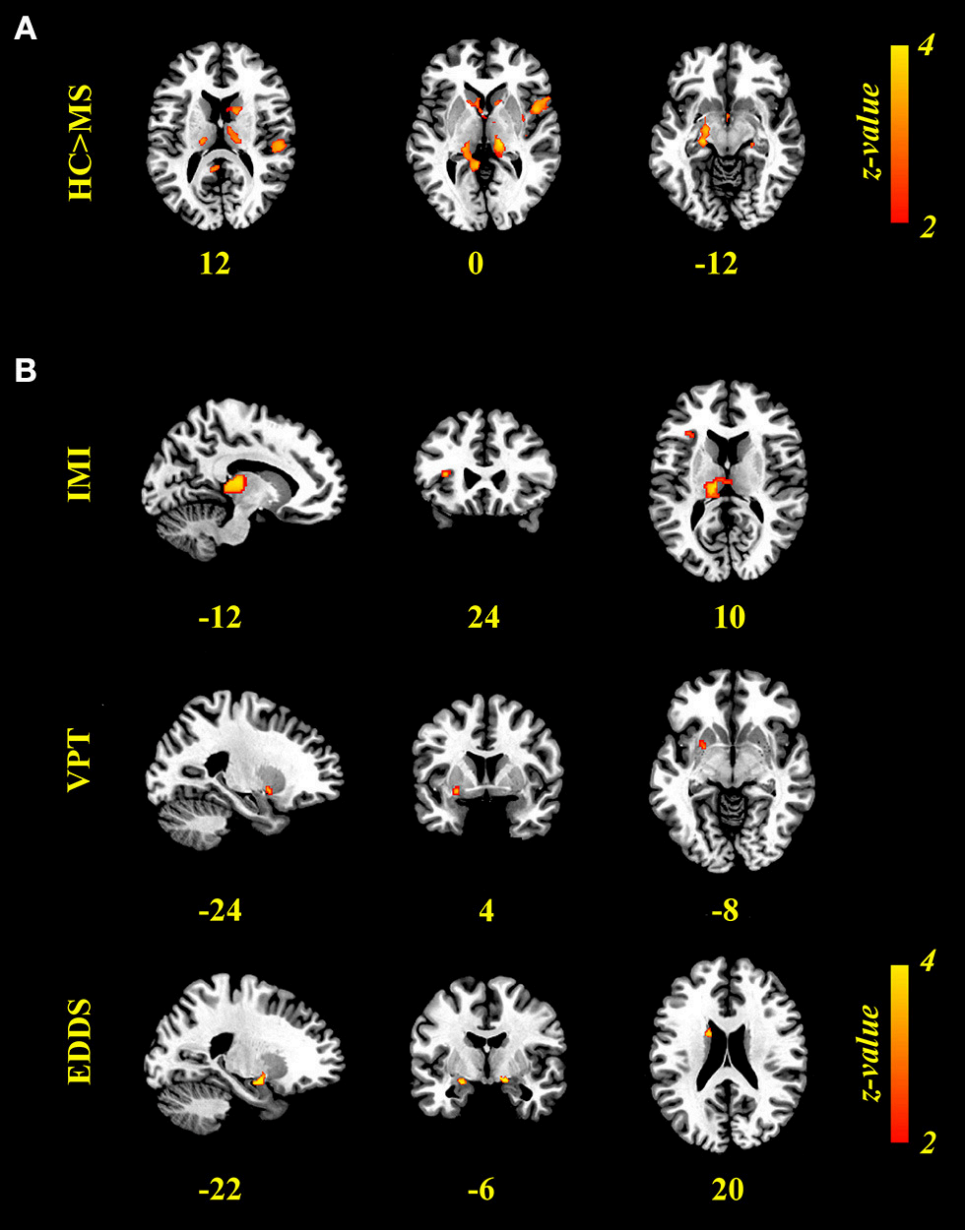
Results of voxel-based morphometry (VBM) analyses. **(A)** Brain regions expressing greater gray-matter volume (GMV) in the HC relative to MS group (*p* < 0.01, FWE-corrected). **(B)** Brain regions in which GMV was associated positively with imitation measured on the Stimulus-response compatibility procedure (IMI) and accuracy of visual perspective taking on the Director Task (VPT), and negatively with clinical scores (EDDS; *p* < 0.05, FW-corrected). VBM results are presented on the Colin template in MNI space, neurological orientation, with values presenting the *x, y*, or *z* coordinate of the corresponding slice.

**Figure 4:**
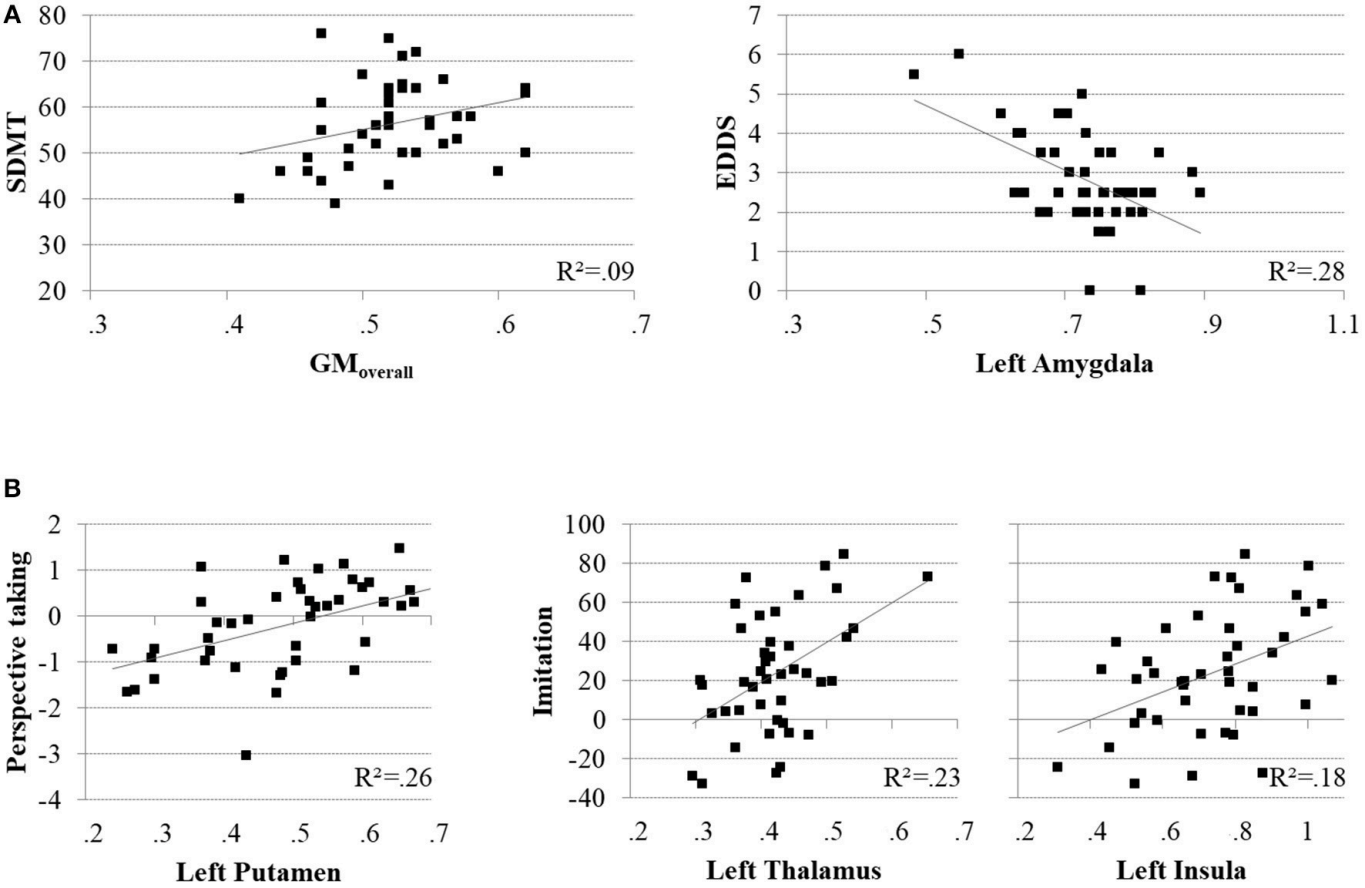
Scatter plots illustrating significant brain-behavior relationships for the MS group. **(A)** Positive association between cognitive processing speed (SDMT) and the overall pattern of relative GMV decline (GM_overall_; left), and negative association between EDSS scores and GMV in the left amygdala (left). **(B)** Positive associations between accuracy in perspective taking and GMV in the left putamen (left), and imitative tendencies and GMV in the left thalamus (middle) and left insula (right).

A whole-brain GLM analysis revealed a diffuse collection of cortical and subcortical regions in which GMV was reduced in MS patients relative to HCs, after accounting for variability in mean WMV (*p* < 0.01, FWE-corrected): this encompassed right lateral temporal cortex and the amygdala; and the bilateral amygdala, caudate nucleus, pallidum, putamen, thalamus, and hippocampus. We refer to this herein as GM_overall_, and these results are presented in Table 2 and Figure 3A. Interestingly, only cognitive processing speed (SDMT scores) was related with GM_overall_–higher processing speed was associated with more GMV throughout this pattern of brain regions (ρ_(41)_ = 0.36, *p* = 0.019; CI = [0.01, 0.63]; see Figure 4A).

**Table 2:** Results of voxel-based morphometry analyses, presenting peak voxels of clusters in which gray matter volume was higher in the HC relative to the MS group (HC > MS; *p*_FWE_ < 0.01), or associated with imitation (IMI), perspective-taking performance (VPT) or clinical characteristics (EDSS; *p*_FWE_ < 0.05).

	**Label**		**# Voxels**	**Peak**	**x**	**y**	**z**
HC > MS	Thalamus	R	10638	5.00	15	−26	3
	Insula/opercular cortex	R	2532	4.52	47	5	1
	Temporal pole	R	1740	4.68	40	10	−32
	Planum temporale	R	1168	4.16	55	−25	13
	Caudate nucleus	L	160	4.06	−20	3	17
	Insula (posterior)	R	25	3.28	40	−16	−2
IMI	Thalamus	L	236	3.73	−10	22	10
	Insula (anterior)	L	5	3.47	32	24	10
VPT	Putamen	L	8	3.23	−26	4	−8
EDSS	Amygdala	L	59	4.02	−22	−4	−14
		R	40	4.54	16	−4	−14
	Caudate	L	40	4.54	16	−4	−14

When adding clinical measures as covariates in GLM analyses of MS patients, neither disease duration nor SDMT scores showed significant associations with localized GMV. Scores on the EDDS, however, were associated negatively with GMV in the bilateral amygdalae and left caudate nucleus (*p* < 0.05, FWE-corrected). In terms of performance on the experimental tasks, accuracy in perspective taking was associated positively with GMV in a small ventral aspect of the left putamen (*N* = 38; *p* < 0.05, FWE-corrected). To investigate relationships between brain structure and behavior on the tasks expressed as response time, we added SDMT scores as an additional covariate of no interest; although we observed no significant relationships between task performance and cognitive processing speed, this allowed us to identify brain-behavior relationships that were independent of general processing speed. Response times in perspective taking showed no significant associations with GMV, but the degree of imitative tendencies demonstrated on the stimulus–response compatibility procedure was associated positively with GMV in a large portion of the left thalamus and left anterior insula cortex (*N* = 43; *p* < 0.05, FWE-corrected). These results are presented in Figure 3B and Table 2, and Figure 4 plots selected significant brain-behavior relationships across the MS sample.

Finally, since our sample varied in achieved education level (see Table 1), we investigated whether this might have influenced the above findings; specifically, we compared both GMV and behavioral performance between the 19 patients with secondary school education and 24 with university degrees. Using the exact same parameters with *randomize*, no significant differences were observed between these two patient subgroups. The same (null) result was obtained when contrasting cognitive, socio-cognitive, and self-report scores (*p* ≥ 0.140).

## Discussion

This study sought to achieve a better characterization of the disruptions to social cognition observed in MS by investigating lower-level facets of socio-cognitive abilities, and examining the neuroanatomical correlates of any impairments. Our findings indicate that, behaviorally, patients with relapse-remitting MS exhibit less involuntarily imitation toward the actions of others compared with HCs, and find it more difficult to adopt another individual's perspective when it differs from their own. Furthermore, this behavioral pattern is associated with reduced GMV in the patient group within deep brain nuclei, revealing a potential neuroanatomical correlate.

To our knowledge, this is the first neurobehavioral investigation of imitative tendencies and visual perspective taking in a neurological population. On the basis of our own and others' previous research, we interpret the pattern of behavior exhibited by our sample of MS patients to reflect impaired self-other distinction [SOD; (4, 16, 24)]. Efficient perspective-taking performance is shown by individuals who can switch flexibly between altercentric and egocentric viewpoints, and report a balanced attentional focus between the self and others during social interactions (55). In contrast, MS patients appear to fall back on a default cognitive state of self-bias when faced with competing self- and other-representations (56)—they appear to be less able to detach themselves from their own self-perspective in order to infer conflicting viewpoints. Likewise, MS patients are less influenced by others' actions. Interestingly, we observed the same pattern of behavior in a large healthy sample; specifically, poorer perspective taking and reduced imitation was observed in individuals characterized by an inflexible personality profile compared with those exhibiting more flexibility (16). In this light, the disturbances presented by MS patients in high-level socio-cognitive capacities, such as mentalizing, might reflect dysfunction to a more fundamental, low-level cognitive mechanism responsible for distinguishing flexibly between self and other representations.

In contrast to previous research (8, 25, 57), MS patients performed equally as well as HCs on a task measuring emotion recognition (RMET). While this might indicate that emotion recognition is preserved in this sample of MS patients, it may simply highlight an important difference between the three experimental tasks we employed; namely, their differential requirements for fast and flexible switching between self and other representations: While successful performance on the Director Task (DT) and Stimulus-response Compatibility (SRC) procedure necessitates swift SOD, the RMET involves a selection of one of four choices describing the mental states expressed by eyes, placing less demands on SOD flexibility. In a similar vein, differential performance on these tasks could result from their different demands on executive function; both the DT and SRC procedure are essentially response inhibition tasks, whereby successful performance necessitates the speeded selection of relevant and suppression of irrelevant information [e.g., (58)]. This is less true of the RMET. Indeed, we suggested that individual differences in cognitive control might underlie the opposing behaviors we observed previously between flexible and inflexible personality profiles (16). It is particularly noteworthy that we also observed equivalent empathic responses between these two profiles. Since the present study did not perform a thorough assessment of executive functioning in MS patients, we are unable to make further claims about its influence of SOD. However, recent research points to the functional independence of executive dysfunction and impairments to social cognition in the MS population [see (59)].

In line with previous research, our measures of social cognition were not associated with self-reported depression (3, 7–9), fatigue (11), or cognitive decline [(8, 11); but see (3)]. This suggests that these frequent symptoms of MS do not predict disturbances in social cognition on their own. Additionally, we observed no relationships between physical disability and our measures of socio-cognitive performance. In contrast, poorer accuracy in perspective taking was associated with disease duration.

Turning now to our neuroanatomical findings, the pattern of GMV reduction we have observed in MS patients aligns closely with previous studies; these structural alterations occurred in the thalamus, putamen, caudate nucleus, globus pallidus, and the amygdala, structures in which GM atrophy frequently appears first and progresses rapidly (26–28). Furthermore, relationships between gray matter and task performance in a selection of these deep brain structures implicate them in the socio-cognitive impairments exhibited by MS patients; reduced GMV was associated with poorer perspective taking in the left putamen, and reduced automatic imitation in the thalami and the left anterior insula (AI), independent of cognitive processing speed. Although the neuroanatomical basis of socio-cognitive disturbances in MS remains understudied (60), these results do converge with studies in the healthy population: First, brain function within the dorsal striatum is reported during the cognitive aspect of mentalizing (29), when differentiating between social actions performed by the self and others, and when processing social behavior in general (61). Second, recent research indicates that intact functioning of thalamus is critical for dynamic integration of information across various cortical networks (62), including those implicated in socio-cognitive and -affective processes [e.g., mentalizing; see (29)]. Interestingly, this region seems to be integral for behavioral flexibility (63)—a role that may extend to flexible self-other switching. Third, previous research has associated brain function within the AI with imitative tendencies (5), as well as processes of interoception, empathy, and social awareness (64). Although the majority of existing studies have linked AI with affective components of social cognition, the left AI has been reported to be activated by both emotional and cognitive aspects (13).

It is noteworthy that all GM areas associated significantly with social cognition were localized primarily to the left hemisphere. Although this observation is in line with other findings reporting a predominantly left-lateralized network of brain regions associated with other aspects of social cognition (65), research into the lateralization of disturbances exhibited by MS patients remains scarce and inconsistent (25); socio-cognitive processes have been related to GMV throughout both left and right hemisphere (33, 65). Interestingly, previous research has indicated that cognitive flexibility is associated with brain regions in the left hemisphere (66), while self-referential processing is linked primarily with the right (67). As such, it might be that our results reflect a selective deficit of cognitive flexibility and preserved self-processing. This finding, then, presents a new avenue of investigation in MS.

Given the potential for functional neuroimaging as an evaluative tool for the early detection of brain alterations in MS, future studies should explore the brain networks engaged by the two experimental tasks we have used to reveal disruptions in SOD. It is important to acknowledge that we have investigated GM volume in what is primarily a WM disorder. While patterns of GM pathology appear to be associated more heavily with cognitive dysfunction in MS than concomitant WM lesions (68), diffuse WM abnormalities are likely to result in the disconnection of brain networks supporting social cognition (32). Interestingly, Plata-Bello et al. (69) report decreased functional connectivity in the brains of MS patients relative to HCs during action observation/execution. Future research should investigate whether the subcortical pattern of GMV reduction that we have observed in MS is related to a loss of WM integrity.

It is important to acknowledge aspects of our study that might limit the generalization of the present results. First, we examined only one index of cognitive functioning—scores on the SDMT. Although this is considered a gold-standard screening tool in MS, and a reliable predictor of cognitive decline in MS (38, 39), we were unable to explore relationships between social cognition and other, more general cognitive domains. As alluded to earlier, the DT and SRC procedure are believed to require response inhibition (58). A more detailed neuropsychological examination of cognitive performance might provide better insights into the relationship between this aspect of executive function and low-level components of social cognition. Second, the group of MS patients recruited in this study differed significantly in their level of educational attainment from those in the HC group. While this difference did not manifest in either behavioral performance or our metric of brain structure, the results of the present study should be treated with caution until they are replicated in comparisons of more educationally balanced MS patients and healthy controls. Third, behavioral assessment was performed 5 months prior to brain scanning on average. While substantial GM atrophy is unlikely to occur during this time interval in MS patients with cognitive reserve (70), it is possible that new WM lesions could have developed. On a related note, because this study recruited a sample of asymptomatic MS patients, a non-routine MRI protocol was performed that involved the acquisition of only T1-weighted images. Lesions of WM appear as hypointensities on T1 images, resulting potentially in an overestimation of GMV. We have attempted to control for this by adding estimates of total WMV calculated from the same T1 image as a covariate of no interest. This is a crude approach, however, and much more accurate methods are available; with the addition of T2-weighted images, automated WM lesion-detection tools (71) can provide accurate estimates of focal lesion load, allowing for lesion masking that improves volumetric estimation [e.g., (25)]. As such, although the brain-behavior relationships we have observed in the present study converge closely with previous research, our findings require replication in future research that addresses these limitations.

In conclusion, our study revealed a potential low-level cognitive mechanism underlying the socio-cognitive disturbances exhibited by patients with MS; at the behavioral level, the performance of MS patients indicated increased self-bias when faced with conflicting self- and other-representations—while emotion recognition seems to be preserved, they showed poorer perspective taking and less involuntary imitation. These selective behavioral impairments were associated with a pattern of reduced GMV that encompasses deep brain nuclei, pointing toward a neuroanatomical correlate for this socio-cognitive profile. Future research should build on our findings by clarifying the influence of structural alterations in these discrete brain structures on SOD, and how this manifests in other socio-cognitive capacities.

## Ethics Statement

The experiment was approved by the Institutional Review Board of St. Anne's University Hospital, and all individuals written informed consent prior to participating.

## Author Contributions

KC: experimental design, behavioral data analyses, manuscript preparation. DJS: experimental design, neuroimaging data analyses, manuscript preparation. KS: recruitment and data collection, manuscript preparation. MD: clinical evaluation, manuscript preparation. RM: neuroimaging data analysis. JV: data collection. MB: experimental design, manuscript preparation.

### Conflict of Interest Statement

The authors declare that the research was conducted in the absence of any commercial or financial relationships that could be construed as a potential conflict of interest.
