# Supplementary material for: Impaired Self-Other Distinction and Subcortical Gray-Matter Alterations Characterize Socio-Cognitive Disturbances in Multiple Sclerosis
Source: Front Neurol. 2019 May 21;10:525. doi: 10.3389/fneur.2019.00525 (PMC6536606; doi:10.3389/fneur.2019.00525)
Supplement: Supplementary file 1 [file Data_Sheet_1.docx]

**Task instructions**

*Stimulus-Response Compatibility Procedure (measure of imitative tendencies; note that color-finger pairing was counterbalanced across participants)*

Press and hold down the <LEFT> and <RIGHT> keys with your index and middle finger, respectively. Release one of the keys as fast as possible when you see a colored dot between the fingers: GREEN = Release <LEFT>, RED = Release <RIGHT>. Press down on both keys again before the next trial begins. DO NOT MOVE unless you see a colored dot. Press <SPACE> to start a practice.

*Director Task (measure of visual perspective taking)*

Here, you can see a shelf displayed from your own perspective [*point to the left-hand shelf*]. You will be given some auditory instructions by the “Director” [*point to the Director*] to move one of the objects to a new location on the shelves. You should move the object according to the Director’s point of view. Since she is sat behind the shelves, she sees things from a different perspective; for example, she will not be able to see some of the objects that you can see [*point to the right-hand shelf*]. All you need to do is click on the destination box – please do not try to move (i.e. drag) the object. You can move items to greyed-out squares; the Director is aware that they are boxes – they just do not know the contents of these boxes. Please try to respond as fast but as accurately as possible.

**Your view: Director’s view:**


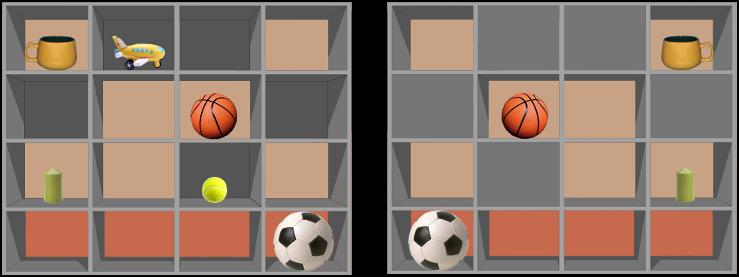


Table S1. Associations between clinical assessment variables and socio-cognitive measures in the MS patient group.

|  |  | BDI | MFIS | SDMT | RMET | IMI | VPT | |
| --- | --- | --- | --- | --- | --- | --- | --- | --- |
|  |  |  |  |  |  |  | RT | Acc |
| DD | *ρ* | -.09 | -.18 | -.22 | -.27 | .26 | .05 | **-.36** |
|  | Sig | .555 | .257 | .165 | .086 | .097 | .749 | **.026** |
|  | CI | [-.37, .21] | [-.46, .15] | [-.49, .13] | [-.55, .06] | [-.04, .51] | [-.26, .36] | [-.62, -.06] |
| EDSS | *ρ* | -.08 | .06 | -.29 | -.15 | -.09 | .18 | -.07 |
|  | Sig | .611 | .705 | .056 | .332 | .564 | .278 | .688 |
|  | CI | [-.37, .20] | [-.24, .32] | [-.56, .05] | [-.42, .15] | [-.39, .23] | [-.16, .50] | [-.39, .25] |

*Abbreviations:* DD = disease duration, EDSS = Expanded disability status scale, BDI = depression, MFIS = fatigue, SDMT = cognitive processing speed, RMET = emotion recognition, IMI = imitative tendencies, VPT = visual perspective taking (RT = response time, Acc = accuracy).

Table S2. Associations between cognitive processing speed and socio-cognitive measures for both groups.

|  | Group |  | RMET | IMI | VPT | |
| --- | --- | --- | --- | --- | --- | --- |
|  |  |  |  |  | RT | Acc |
| SDMT | MS | *ρ* | .19 | .11 | .13 | .13 |
|  |  | Sig | .217 | .501 | .425 | .441 |
|  |  | CI | [-.09, .47] | [-.23, .40] | [-.19, .47] | [-.23, .49] |
|  | HCs | *ρ* | -.05 | -.02 | -.11 | .20 |
|  |  | Sig | .753 | .926 | .488 | .219 |
|  |  | CI | [-.35, .26] | [-.34, .32] | [-.41, .19] | [-.11, .49] |

*Abbreviations:* MS = patient group, HCs = healthy controls, SDMT = cognitive processing speed, RMET = emotion recognition, IMI = imitative tendencies, VPT = visual perspective taking (RT = response time, Acc = accuracy).
